# Patients with mild and general COVID-19 should be negative for at least 3 consecutive nucleic acid tests before discharged

**DOI:** 10.1371/journal.pone.0240081

**Published:** 2020-10-02

**Authors:** Rui Lu, Tianhui Huang, Haiqing Hu, Xiao-Ping Liu

**Affiliations:** 1 Department of Orthopedics, Wuhan Third Hospital, Tongren Hospital of Wuhan University, Wuhan, PR China; 2 Department of Traditional Chinese Medicine, Wuhan Third Hospital, Tongren Hospital of Wuhan University, Wuhan, PR China; 3 Department of Urology, Zhongnan Hospital of Wuhan University, Wuhan, PR China; Universidad Nacional de la Plata, ARGENTINA

## Abstract

Given the global spread of coronavirus disease (COVID-19), strict discharge standard is of great significance for the prevention and control of the epidemic, thus, the purpose of this study is to formulate more strict and scientific discharge standards. A total of 845 patients with mild and general COVID-19 who were considered to be discharged from hospital were included in this study. The median time from the onset of COVID-19 to the occurrence of two consecutive negative nucleic acid tests of these patients was 21 days. 223 of the 845 patients were tested again after two consecutive negative nucleic acid tests and 17.49% of the patients were positive. Moreover, 82.51% (184 of 223) of these patients experienced negative results from three consecutive nucleic acid tests, the median time from the onset of COVID-19 to the occurrence of three consecutive negative nucleic acid tests was 23 days (range: 3–56 days), and 38 of which were further tested after three consecutive negative nucleic acid tests, while about 5.26% (2 of 38) patients showed positive nucleic acid test results. Thus, we suggested that the patient should be negative for at least 3 consecutive nucleic acid tests before discharge, and the test time should be no earlier than the 23rd day since the onset of the disease.

## Introduction

Since December 2019, a new coronavirus disease (COVID-19) epidemic has occurred in Wuhan, Hubei Province, China. With the spread of the epidemic, COVID-19 has appeared in various provincial administrative units and special administrative regions in China. In addition, COVID-19 has occurred in many countries around the world, leading to the World Health Organization (WHO) made the assessment that COVID-19 could be characterized as a pandemic [1]. Through a series of preventive control and medical treatment measures across the country, the rising trend of the epidemic in China has been effectively contained. As of 24:00 on April 8, 2020, there were 81,865 confirmed diagnoses in China, and a total of 77,370 patients were discharged from the hospital. The number of new cases per day has fallen below 100 [2]. According to the latest COVID-19 clinical diagnosis and treatment guideline [3], patients who meet the following four criteria can be discharged: (1) the body temperature has returned to normal for more than 3 days; (2) the respiratory symptoms have improved significantly; (3) the pulmonary imaging examination shows that the acute exudative lesions have improved significantly, (4) two consecutive negative nucleic acid test for respiratory specimens such as sputum and nasopharyngeal swabs (sampling interval at least 24 hours). However, based on our clinical observations and experience, some COVID-19 patients were tested again for COVID-19 nucleic acid when they met the above four discharge criteria, and the results still had a certain positive rate. The clinical observations of this research team are reported as follows.

## Methods

**Fig 1:**
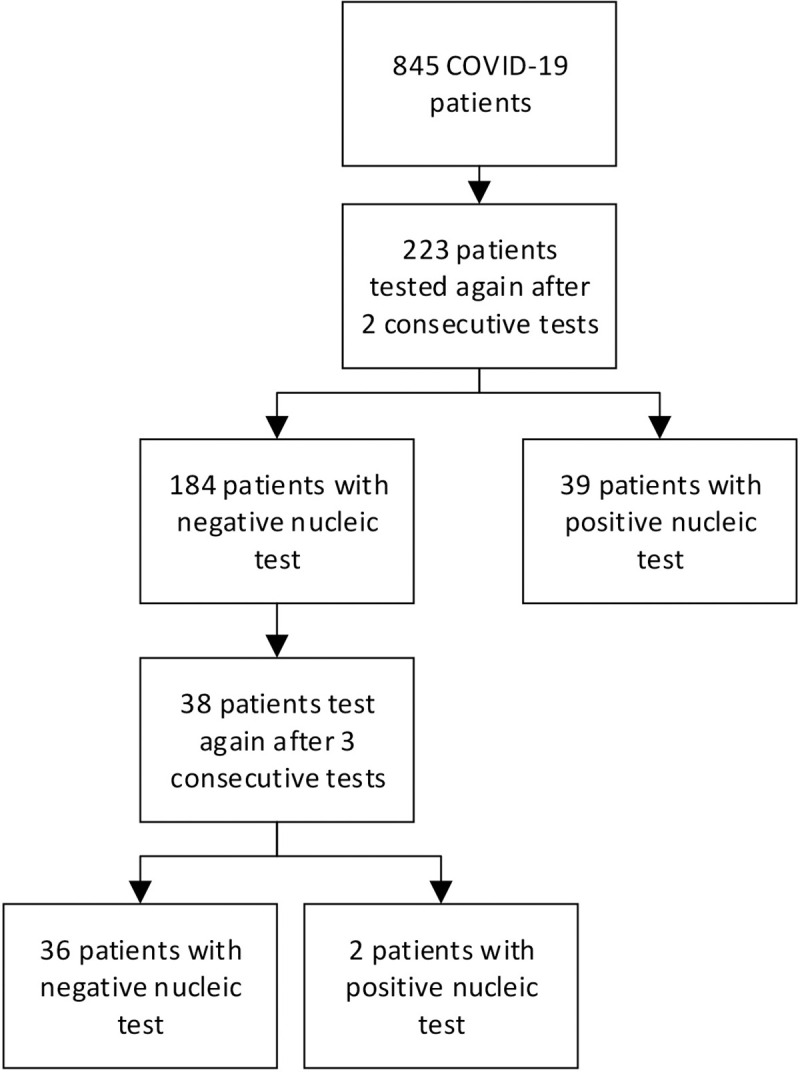
The flow chart depicting the composition of COVID-19 patients at different stages.

This retrospective study investigated mild and moderate COVID-19 patients admitted to the Wuchang Cabin Hospital (Wuhan, China) from February 6, 2020 to March 9, 2020. Wuchang Cabin Hospital is a hospital temporarily reconstructed from Hongshan Gymnasium to solve the "one bed is difficult to find" Wuhan Crisis. It opened the earliest and shut down latest in Wuhan. Patients met the following inclusion criteria were included in this study: (1) patients with mild and general COVID-19 [3]; (2) Patients with complete self-care ability; (3) patients diagnosed with COVID-19 through nucleic acid test. Meanwhile, patients who met the following exclusion criteria were excluded: (1) patients with severe diseases of severe respiratory system, cardiovascular and cerebrovascular systems; (2) patients with mental illness or cognitive impairment; (3) patients with a positive influenza virus test; (4) patients who need to be transferred due to worsening condition during hospitalization. All patients received unified antiviral (arbidol, oseltamivir, ribavirin), antibiotic (moxifloxacin), traditional Chinese medicine (Lianhuaqingwen capsule) and immunomodulatory (interferon, thymopolypeptides) treatment. After the patient met the first three of the above discharge criteria, we began to perform continuous SARS-CoV-2 nucleic acid testing on the patient using RT-PCR. Nucleic acid kits were provided by Wuhan COVID-19 Control Command. The interval from the onset of COVID-19 to two consecutive negative results of nucleic acid test was recorded, as well as the probabilities of recurrence of a positive nucleic acid test after two and three consecutive negatives. The data of onset of symptoms, admission date, nucleic acid testing date, and discharge date were collected from electronic medical records. The flow chart depicting the composition of patients at different stages was presented in Fig 1.

Description analysis was conducted to reveal the demographic characteristics (gender and age) using the R package “table1” (https://cran.rstudio.com/web/packages/table1/). Chi-square test and t test were used to determine the differences gender and age distributions between different groups, respectively. This study was approved by the Institutional review board of the Wuhan Third Hospital and the need for informed consent was waived.

## Results

A total of 845 patients were included in this study, including 369 (43.7%) males and 476 (56.3%) females. The median age of the 845 COVID-19 patients was 49 years (range: 9–82 years). The dates of onset of symptoms of these patients were ranging from December 31, 2019 to February 24, 2020. The admission dates of these patients were ranging from February 6, 2020 to February 26, 2020. The dates of nucleic tests were ranging from January 15, 2020 to March 9, 2020. The discharge dates were ranging from February 13, 2020 to March 10, 2020.

The median time from the onset of COVID-19 to the occurrence of two consecutive negative nucleic acid tests in 845 patients with COVID-19 was 21 days (range: 1–56 days). The median interval between the two nucleic acid tests was 2 days (range: 1–33 days). In the total 845 COVID-19 patients, 223 patients were tested again after two consecutive negative nucleic acid tests, the characteristics of the 233 patients were summarized in Table 1. The median interval between the two consecutive nucleic acid tests and the further nucleic acid test was 3 days (range: 1–14 days). The positive probability of retesting after two consecutive negative nucleic acid tests was 17.49%.

**Table 1:** The characteristics of COVID-19 patients who received further nucleic acid test after 2 consecutive negative nucleic tests.

Variables	Negative	Positive	P value
	(n = 184)	(n = 39)	
**Age (Years)**			
Mean (SD)	48.8 (12.5)	48.8 (12.5)	0.993
Median [Min, Max]	51.0 [11.0, 79.0]	53.0 [27.0, 69.0]	
Missing	2 (1.1%)	0 (0%)	
**Gender**			
Male	79 (42.9%)	16 (41.0%)	0.925
Female	103 (56.0%)	23 (59.0%)	
Missing	2 (1.1%)	0 (0%)	

Next, we tried to investigate the positive probability of retesting after three consecutive negative nucleic acid tests. A total of 184 patients experienced negative results from three consecutive nucleic acid tests, the median time from the onset of COVID-19 to the occurrence of three consecutive negative nucleic acid tests was 23 days (range: 3–56 days), and 38 of which were further tested after three consecutive negative nucleic acid tests, while about 5.26% (2 of 38) patients showed positive nucleic acid test results.

## Discussions

As mentioned above, the outbreak of COVID-19 is showing a continuous improvement in China. Nearly a thousand patients are discharged from hospital every day, but in some countries outside China, there is an outbreak trend. At the same time, China also faces the risk of overseas COVID-19 import. As we know, COVID-19 is a highly contagious infectious disease, thus, strictly controlling the discharge criteria of patients is of great significance to the prevention of community rebound and spread of the disease. There is a shortage of nucleic acid testing reagents in some countries. Therefore, scientific and reasonable nucleic acid testing time and intervals are of great significance for patients diagnosed with COVID-19 to receive timely treatment, early isolation and control of the source of infection, to which avoid further spread of the epidemic.

This study showed that even if the patient meets the discharge standard of body temperature, respiratory symptoms, imaging findings, and after two consecutive negative nucleic acid tests, 17.49% of patients were positive for nucleic acid tests (that is, the coronavirus had not been completely eliminated in the patient), consistent with previous reports [4]. Thus, it could not exclude the possibility that COVID-19 is a disease similar to the hepatitis B whose virus cannot be completely cleared in the body, which was worth our vigilance [5]. Meanwhile, if COVID-19 patients who met other discharge criteria were tested for 3 consecutive nucleic acids (median test time was 23 days), the positive rate of nucleic acid test after three nucleic acid tests might be 5.26%. Therefore, we suggest that 3 consecutive nucleic acid tests should be given to patients when they are considered to be discharged. The test should be performed no earlier than the 23rd day after the onset of symptoms and the clinical performance has improved significantly after formal treatment.

This study is a retrospective study, so it is inevitable that there are certain defects. First of all, there were a number of patients in this study who did not receive further nucleic acid tests after 2 and 3 consecutive negative nucleic acid tests. This may be due to the insufficient understanding of the phenomenon of positive nucleic acid test after 2 consecutive negative nucleic acid tests in the early stage of the epidemic. The lack of patient data in this section directly reduced our sample size to assess the probability of the nucleic acid test returning positive after two consecutive negative nucleic acid tests. In this regard, we will further improve the follow-up of patients, and their outcomes have been clarified.

In summary, we recommend that when the patient is scheduled to be discharged from hospital, the patient should be negative for at least 3 consecutive nucleic acid tests, and the test time should be no earlier than the 23rd day since the onset of the disease.
